# Tricuspid Regurgitation – Medical Management and Evolving Interventional Concepts

**DOI:** 10.3389/fcvm.2018.00049

**Published:** 2018-05-28

**Authors:** Frederik Beckhoff, Brunilda Alushi, Christian Jung, Eliano Navarese, Marcus Franz, Daniel Kretzschmar, Bernhard Wernly, Michael Lichtenauer, Alexander Lauten

**Affiliations:** ^1^Department of Cardiology, Charité University Hospital, Berlin, Germany; ^2^German Centre for Cardiovascular Research (DZHK), Berlin, Germany; ^3^Department of Cardiology, Pulmonology and Vascular Medicine, Medical Faculty, University Düsseldorf, Düsseldorf, Germany; ^4^Interventional Cardiology and Cardiovascular Medicine Research, Inova Center for Thrombosis Research and Drug Development, Inova Heart and Vascular Institute, Falls Church, VA, United States; ^5^SIRIO MEDICINE Network, Evidence-Based Section, Falls Church, VA, United States; ^6^Cardiovascular Institute, Ludwik Rydygier Collegium Medicum, Nicolaus Copernicus University, Bydgoszcz, Poland; ^7^Department of Internal Medicine, Friedrich-Schiller-University Jena, Jena, Germany; ^8^Department of Cardiology, Internal Medicine II, Paracelsus Medical University Salzburg, Salzburg, Austria

**Keywords:** Tricuspid Valve Regurgitation, Interventional Therapies, The FORMA Device, Trialign, TriCinch, Traipta, Orthotopic Valve Replacement, Heterotopic Valve Replacement

## Abstract

Severe tricuspid regurgitation (TR) is a complex condition of the right ventricle (RV) and tricuspid valve apparatus and is frequently associated with symptomatic heart failure and a significant morbidity and mortality. In these patients, left heart pathologies lead to chronic pressure overload of the RV, eventually causing progressive RV dilatation and functional TR. Therefore, TR cannot be considered as isolated heart valve disease pathology but has to be understood and treated as one component of a complex structural RV pathology and is frequently also a marker of an advanced stage of cardiac disease. In these patients, medical therapy restricted to diuretics and heart failure medication is frequently ineffective. Also, severe TR in the setting of advanced heart failure constitutes a high risk for cardiac surgery. Neither one of these treatment options has demonstrated a beneficial effect on long-term prognosis. The recent innovations in transcatheter technology led to efforts to develop interventional approaches to severe TR. Multiple innovative treatment concepts are currently under preclinical and clinical investigation to replace or repair TV function. However, up to date none of these approaches is established and there is still a lack of clinical data to support the efficacy of transcatheter TR treatment.

## 1. Introduction

Severe tricuspid regurgitation (TR) is a complex condition of the right ventricle (RV) and tricuspid valve apparatus and is frequently associated with symptomatic heart failure and a significant morbidity and mortality (1, 2). In these patients, left heart pathologies lead to chronic pressure overload of the RV, eventually causing progressive RV dilatation and functional TR (3–7). Therefore, TR cannot be considered as isolated heart valve disease pathology but has to be understood and treated as one component of a complex structural RV pathology and is frequently also a marker of an advanced stage of cardiac disease.

In these patients, medical therapy restricted to diuretics and heart failure medication is frequently ineffective. Also, severe TR in the setting of advanced heart failure constitutes a high risk for cardiac surgery (8–10). Neither one of these treatment options has demonstrated a beneficial effect on long-term prognosis. The recent innovations in transcatheter technology led to efforts to develop interventional approaches to severe TR. Multiple innovative treatment concepts are currently under preclinical and clinical investigation to replace or repair TV function (11–17). However, there is still a lack of clinical data to support the efficacy of transcatheter TR treatment. Up to date, none of these approaches gained a CE mark or FDA approval and therefore they are only meant for investigational purposes.

## 2. Anatomy of the Tricuspid Valve and the Right Ventricle

The tricuspid valve anatomy is very complex and has a greater variability than the mitral valve. It is composed of the fibrous annulus, three leaflets, three papillary muscles, and the chordae tendineae. It is embedded in the right ventricular inflow tract in the junction between the right atrium (RA) and the right ventricle (RV). The elliptical shaped fibrous annulus is a three-dimensional, highly dynamic structure. It is slightly larger and anatomically not as well defined as the mitral annulus. Its size and shape changes during the cardiac cycle due to contraction of the surrounding myocardium (18). The annulus provides a base for the prominent anterior, the septal and a mostly hypoplastic posterior leaflet. Valve calcification is a pathologic process but the valves are less prone to calcification compared to the mitral valve even at an advanced disease state and age. During right ventricular contraction, the annular area decreases by approximately 25–30%, which is essential for leaflet coaptation and competence of the valve (19–21). Thus, any conditions altering geometry of the tricuspid valve apparatus - such as changing loading conditions or structural enlargement of the right-sided chambers - negatively impact valve function. Adjacent to the attachment of the septal leaflet there is the AV-node and the right coronary artery encircles the valve.

## 3. Epidemiology and Prognostic Impact of TR

TR (TR) is a common insufficiency typically graded in mild, moderate and severe TR. While mild TR is frequently observed in asymptomatic persons, moderate and severe TR are seen less often (22). As even with pronounced TR many patients remain asymptomatic for several years the assessment of prevalence is difficult. In the US population, moderate-to-severe TR was estimated at approximately 1.6 million cases (23, 24).

However, as TR frequently develops with the progression of left heart or pulmonary disease, the underlying disorder rather than the tricuspid valve lesion tends to dominate the clinical picture. Increased right atrial pressure is transmitted to the central and hepatic veins leading to hepatosplenomegaly and ascites, which are present in 90% of patients with severe TR (23, 24).

Nath et. al conducted an echocardiographic series of 5,223 patients. Moderate to severe TR was found in 16% of patients (1). With increasing severity of TR mortality increased. The poor prognosis of the severe TR is independent of left ventricular ejection fraction and pulmonary hypertension (1). Several more recent studies confirmed these results and therefore underlined the correlation between severity of TR and increased mortality (25–29). In addition, patients with severe symptomatic TR showed prolonged hospitalization and a higher rate of rehospitalization (26).

## 4. Pathophysiology

TR is most frequently “functional” in nature (8). It is commonly observed in patients with left heart valve disease, myocardial disease or pulmonary hypertension. A volume overload or/and elevated RV pressure leads to a right ventricular (RV) remodeling with RV enlargement followed by a tricuspid annulus dilatation. An increase in TV annular area develops primarily due to dilatation of the posterior and lateral segments along the right ventricular free wall (30, 31). Another mechanism of functional TR if an impaired leaflet function e.g., as in the presence of a lead crossing the TV.

In early stages RV and annulus dilatation can still be compensated and TR is not severe. A progressive dilatation leads to papillary muscle displacement and tethering of the leaflets causing severe TR. Regurgitation volume leads to increased diastolic loading which supports further RV and annulus dilatation entering a vicious circle Because right ventricular stroke volume is partially expelled backwards into the venous system, there is a resulting decrease in cardiac output and RV afterload. This decrease in right ventricular afterload in presence of TR may initially actually mask a decreased RV contractility. As right ventricular preload rises the right ventricle loses its contractility and eventually fails. As the tricuspid valve loses it function, hemodynamic parameters of the right atrium adjust to those in the right ventricle called a ventricularisation of the right atrium and finally there is a systolic backflow in the hepatic, abdominal and peripheral veins too.

Primary or non-functional TR is seen less frequent. It occurs when there is damage to the tricuspid leaflets, chordae, papillary muscles, or annulus, independent of right ventricular dysfunction or pulmonary hypertension. It can be caused by infective endocarditis, congenital disease like Ebstein anomaly or atrioventricular canal, rheumatic fever, carcinoid syndrome, endomyocardial fibrosis, myxomatous degeneration of the tricuspid valve leading to prolapse, penetrating and non-penetrating trauma, and iatrogenic damages during cardiac surgery, biopsies, and catheter placement in right heart chambers (3, 32). These entities lead to billowing valve, a prolapse or a flail tricuspid valve.

## 5. Diagnosis of TR

### 5.1. Echocardiography

Echocardiography is the main tool to confirm the diagnosis, determine aetiology and assess the severity of TR. Color flow imaging can be used as a screening method. Grading of the severity should be performed in accordance to the guidelines published in 2010 by the European Society of Echocardiography and in 2017 by the American Society of Echocardiography (32, 33).

These guidelines recommend several structural, qualitative, semiquantitative and quantitative parameters (Table 1). A simple parameter that is easy to obtain and well validated is the vena contracta width (VCW). It is usually obtained in the four-chamber view using color flow imaging. The VCW is measured perpendicular to the commissural line at the narrowest portion of the jet reflecting the regurgitant orifice area. A VCW > 7 mm indicates a severe TR. Lower values are difficult to interpret and therefore should not be used to distinguishing moderate from mild or severe TR. Using a continuous wave (CW) Doppler in the four-chamber view at the tricuspid leaflet tips the TR can be further evaluated. In case of a severe TR the WC signal is intense, truncated and triangular indicating an elevated right atrial pressure. The peak E velocity representing the early diastolic filling increases in the proportion to the degree of TR. A peak E Velocity >1 m/s suggests a severe TR. The proximal isovelocity surface area (PISA) is measured in the apical four chamber view and in the parasternal long axis. Nyquist limit must be lowered to 28 cm/s. PISA radius is than measured using the first aliasing. A PISA radius >9 mm indicates a severe TR whereas a radius <5 mm indicates a mild TR. After calculating the PISA the effective regurgitant orifice area (EROA) and regurgitations volume (Rvol) can be calculated using the time velocity integral in the continuous wave Doppler (CW) signal. An EROA ≥40 mm2 or a Rvol ≥45 mL indicates severe TR.

**Table 1:** Grading the severity of TR.

	Mild	Moderate	Severe
**Structural**			
**TV morphology**	Normal or mildly abnormal leaflets	Moderately abnormal leaflets	Flail leaflet, large coaptation defect, severe retraction, large perforation
**RV size**	Normal	Normal or mild dilatated	Dilatated
**RA size**	Normal	Normal or mild dilatated	Dilatated
**VCI diameter (cm)**	<2	2–2,5	>2
**Qualitative Doppler**			
**Color flow jet area**	Small, narrow, central	Moderate central jet	Very large central jet or eccentric wall impinging jet
**Flow convergence zone**	Not existent	Intermediate in size and duration	Large throughout systole
**CW signal of jet**	Faint/partial/parabolic	Dense, parabolic or triangular	Dense, triangular with early peaking (peak ,2 m/s in massive TR)
**Semiquantitative**			
**Color flow jet area (cm^2^)**	Not defined	Not defined	>10
**VCW (cm)**	<0.3	0.3–0.69	≥0.7
**PISA radius (cm)**	≤0.5	0.6–0.9	>0.9
**Hepatic vein flow**	Systolic dominance	Systolic blunting	Systolic flow reversal
**Tricuspid inflow**	A-wave dominant	A-wave dominant	E-wave >1.0 m/sec
**Quantitative**			
**EROA (cm^2^)**	<0.20	0.20–0.39	≥0.40
**R Vol (ml)**	<30	30–44	≥45

Based on the 2010 guidelines by the European Society of Echocardiography and the 2017 guidelines by the American Society of Echocardiography (32, 33).

CVI, vena cava inferior; CW, continuous-wave; EROA, effective regurgitant orifice area; R Vol, regurgitant volume; RA, right atrium; RV, right ventricle; TR, tricuspid regurgitation; TV, tricuspid valve; VCW, vena contracta with.

In moderate to severe TR evaluation of the RV and RA dimensions, RV function and the venous congestion is mandatory. The tricuspid annular plane systolic excursion (TAPSE) is easy to obtain and well validated to assess the right heart function. A TAPSE <17 mm is highly suggestive of RV systolic dysfunction.

The right heart dimensions have previously been published in an update from the American Society of Echocardiography and the European Association of Cardiovascular Imaging (34). Measurements should be performed in multiple acoustic windows but the most important view is the four-chamber view focused on the RV. In this view, the left apex is in the center of the scanning sector and the largest basal RV diameter is displayed. In this view, a diameter >41 mm at the base and >35 mm at the midlevel in the RV-focused view indicates RV dilatation. The RV longitudinal diameter ranges between 59–83 mm, and therefore a dilatation is present if the longitudinal diameter exceeds 83 mm. Normal diastolic tricuspid valve annulus diameter is 22–33 mm and a significant annulus dilatation is present when the diameter exceeds 21 mm/m^2^ (>40 mm).

The right atrium is assessed in the same echocardiographic window. Mostly performed are linear and volume measurements. Linear measurements include the minor and the major axis of the LA. The minor axis is should be measured as the distance between the lateral RA wall and interatrial septum in the midatrial level. A normal value is 1.9 cm/m^2^. The major axis is orthogonal to the minor axis and is normally between 2.4–2.5 cm/m^2^. The more accurate RA volume is 21–25 mL/m^2^.

The atrial pressure can be estimated by the use of vena cava diameter and the percentage of decrease of diameter during inspiration. An IVC diameter <2.1 cm with a collapse >50% during inspiration suggests normal RA pressure of 3 mm Hg, whereas IVC diameter >2.1 cm that collapses <50% during inspiration suggests high RA pressure of 15 mm Hg (35). In cases where IVC diameter and collapse do not fit this paradigm, an intermediate value of 8 mmHg should be assumed (34). Hepatic veins are an additional tool grading the TR. Using color flow or pulsed wave Doppler (PW) a systolic flow reversal can be observed in patients with severe TR.

## 6. Current Therapy and Its Limitations

### 6.1. Currently Recommended Treatment and Their Indications

Currently medical and surgical therapy are the only established treatments options for patients with severe TR. Medical treatment is considered a symptomatic therapy limited to diuretics. Surgical repair or replacement is associated with a significant mortality and is therefore restricted to a selected group of patients with a suitable risk profile. Also, the timing for surgery remains controversial (36). According to the ESC/EACTS guidelines, patients with primary TR should undergo surgery even when still asymptomatic or when undergoing surgery for left-sided valvular heart disease. In contrast, secondary severe TR is only surgically corrected when patients undergo left-heart surgery. However, these guideline recommendations are Level C recommendations due to the lack of data. Although these patients respond well to diuretic therapy medical treatment should not delay surgery to avoid secondary damage in terms of irreversible RV dysfunction which is associated with worse outcome.

### 6.2. Surgical Therapy

Today surgical correction of TR focuses on restoring valve function by reducing annular size. However, the efforts to achieve the most durable results have resulted in an ongoing debate as to whether annular plication should be achieved by implanting either flexible or rigid rings rather than by means of a partial purse string suture technique (“DeVega-technique”). Rigid annuloplasty rings appear to have a lower incidence of recurrent TR than flexible devices or the DeVega technique. In addition, the edge-to-edge tricuspid valve repair has been suggested as providing an effective adjuvant procedure for severe residual TR following annuloplasty (37, 38). The procedure is analogous to mitral valve repair by leaflet approximation and involves anchoring the anterior leaflet to the facing edges of the septal and posterior leaflets of the tricuspid valve, thus creating a triple orifice. Prosthetic TV replacement is reserved for advanced structural valve disease and carries a higher perioperative risk compared to valve repair. Following tricuspid valve replacement, a lower 10 year survival rate has been reported compared to TV replacement (37–4.8% versus 47.5–3.5%) (39).

### 6.3. Current Interventional Approaches

**Figure 1:**
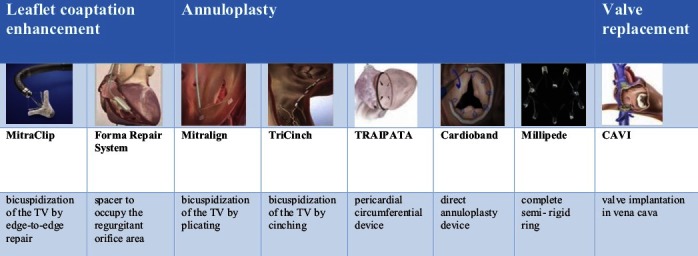
Overview over new tricuspid valve repair devices. CAVI, caval valve implantation; TV, tricuspid valve; TRAIPTA, transatrial intrapericardial tricuspid annuloplasty.

Patients with severe TR are frequently considered inoperable due to comorbidities and surgery is therefore often refused (40, 41). Thus, there is a large unmet need for less invasive treatment options. Recently, the tricuspid valve is receiving much attention from interventional cardiologists and industry, seeking to develop novel catheter-based approaches to TR. So far most of these treatments have been applied in compassionate human cases or small feasibility trials and limited experimental data have been published. Transcatheter TR repair concepts are focusing to reproduce surgical concepts such as annuloplasty or leaflet adoption, but also alternative approaches are under investigation such as coaptation enhancement or heterotopic caval valve implantation (Figure 1).

#### 6.3.1. Leaflet Enhancement

##### 6.3.1.1. The FORMA Device (Edwards Lifesciences)

The FORMA Repair System (Edwards Lifesciences) has been designed for patients with a severe functional TR. Annulus dilatation leads to malcoaptation of leaflets and therefore to a central regurgitant jet. By advancing a spacer in the center of the regurgitant yet the regurgitant orifice area is occupied and leaflets have a new coaptation surface. Therefore, the TR Grade can be reduced. The spacer is a foam-filled polymer balloon that passively expands via holes in the spacer shaft. It is available in 12 and 15 mm both with a length of 42 mm. Two radiopaque markers help to initially position the spacer using fluoroscopy. The distal end is connected to a rail anchored at the apex of the RV whereas the proximal end is locked in the subclavian region with the extra rail length coiled into a pre-pectoral pocket.

The first in men experience with the FORMA device was published in 2015. Seven high-risk patients with severe TR presenting with clinical signs of heart-failure deemed unsuitable for surgery received the FORMA device as a compassionate use. All patients had a New York Heart Association (NYHA) functional class II to IV. Device implantation was successful without procedural complications in all patients. Severity of TR could be reduced significantly to moderate TR in 3 patients and to mild TR in 4 patients. At 30 day follow-up, all patients but 1 demonstrated an improvement in NYHA functional status to class II. No complications related to the device or vascular access were observed during follow-up (42). Recently the one-year experience from eighteen patients at 3 centers in Canada and Switzerland with the transcatheter FORMA system was published. They presented a good mid-term safety profile. Despite variable success in reducing echocardiographic TR grade, there were significant clinical improvements and reductions in right ventricular dimensions (43).

The FORMA system is an investigational device and not for sale in any country. An Early Feasibility Study is currently recruiting patients in the USA (NCT02471807). The 30 day data of the US feasibility trial were presented at the TCT 2017 by Susheel Kodali, MD (NewYork-Presbyterian/Columbia University Medical Center, New York, NY). 30 patients with severe symptomatic TR from five US centers underwent implantation of the Forma device. All in all, the results proved that the device is a feasible therapy for patients with severe TR. Nevertheless, it was associated with infrequent distal anchor dislodgements and the complications thereof. The next generation of this device probably with a more predictable anchor capture without dislodgement or RV perforations is already under development.

Beside the Early Feasibility Study in the USA other multicenter trials are planned in Europe and Canada.

##### 6.3.1.2. MitraClip in Tricuspid Position

The MitraClip® system has been invented for high risk patients with mitral regurgitation which are unsuitable for surgery. It is a procedure that involves the percutaneous implantation of one or more clips grasping and approximating the edges of the leaflets at the origin of the regurgitant jet and therefore reducing it. In 2011, this procedure was presented first time by Feldman et al (44). Until today this has now been integrated in the clinical routine for patients with severe mitral regurgitation at high risk constellation. In 2016, the first clips could successfully be implanted at the tricuspid valve (45). In 2017, Nickenig et al demonstrated the feasibility and safety of leaflet coaptation using the MitraClip in tricuspid position in 64 consecutive patients (46). In this report clips where implanted in multiple commissural sites, while now the strategy is to perform a bicuspidization of the tricuspid valve by a progressive clipping of the septal and anterior leaflet until satisfactory reduction of TR is achieved as well as the gradient across the valve is not increased significantly.

Nevertheless, clipping the tricuspid valve is more difficult than performing a clip in mitral position. The angle between the IVC and the TV annular plane makes coaxial positioning difficult. A transjugular approach has been described but the femoral approach was favored in the above-mentioned study.

#### 6.3.2. Annuloplasty

##### 6.3.2.1. Trialign

The Trilign device (Trialign, Tewksbury, Massachusetts) is a percutaneous minimal invasive annuloplasty system initially designed for the treatment of symptomatic functional mitral regurgitation. First experiences have been made with the treatment of patients with severe tricuspid regurgitation. It was inspired by the Kay bicuspidization procedure, in which the annulus is plicated along its muscular part adjacent to the posterior leaflet (47). With the help of an 8 F articulating wire delivery catheter and a pledget delivery catheter using a transjugular venous approach, pledgets with sutures are fixed at the anteroposterior and septoposterior commissures. These steps are guided by TEE and fluoroscopy. Afterwards a plication lock device is used to bring the 2 pledgeted sutures together, plicating the annulus and effectively bicuspidizing the tricuspid valve.

The first-in-man procedure was published in 2015. The Trialign device was implanted in an 89-year-old woman with right heart failure due to TA dilation and severe TR as a compassionate use. After procedure, there was a significant reduction of tricuspid annulus area (57%) and effective regurgitant orifice area (53%). Hemodynamic parameters in terms of right atrial pressure and left ventricular stroke volume also improved (48). There are two ongoing early feasibility studies, one in the USA (NCT02574650) and one in Europe (NCT03225612). The 30 day results of early feasibility trial in the USA were recently published. The results confirmed the safety of the novel transcatheter device, which reduced tricuspid annulus diameter, effective regurgitant orifice area, increased the left ventricular stroke volume, and improved quality-of-life measurements (49).

##### 6.3.2.2. TriCinch

The TriCinch System (4Tech Cardio Ltd., Galway, Ireland) is a percutaneous device designed to reduce functional TR by reducing the septo-lateral distance. It is composed of two parts: a stainless-steel corkscrew and a self-expanding nitinol stent. Both are connected by a Dacron band. First, the corkscrew is delivered by a delivery system from the Inferior Vena Cava and fixated at the anteroposterior TV annulus. After the delivery system is removed a second delivery system is advanced containing the other half of the TriChinch System. Both Dacron bands are now connected with a locking mechanism. By pulling the TriChinch System tension is applied to the TVA and therefore the septo-lateral distance is reduced. If echocardiography shows the desired reduction of TR, the stent is deployed in the inferior vena cava to maintain the tension.

Four stent sizes are currently available (27, 32, 37, and 43 mm).

The “first-in-man” procedure was reported in 2015. The patients was a 72-year-old woman enrolled in the PREVENT (Percutaneous Treatment of Tricuspid Valve Regurgitation With the TriCinch System; NCT02098200) study. She had a severe functional TR associated with tricuspid annular dilation causing multiple hospitalization. New York Heart Association functional class was III. The procedure was completed in 56 min and the patient was discharged 5 days later. No intra- or postprocedural complications were observed. TR grade could be reduced from grade 4 to grade 3. The 6 month follow-up showed an improved quality of life.

At this time, enrollment of 24 patients in the PREVENT Trial is completed. Results are not published yet.

##### 6.3.2.3. TRAIPATA

The transatrial intrapericardial tricuspid annuloplasty (TRAIPTA) is a minimal invasive device for patients with functional TR. Once implanted it acts as a epicardial annuloplasty band. After a transfemoral venous approach pericardial access is obtained by puncturing the right atrial appendage.

Once the delivery device is advanced in the epicardial space it opens a preformed loop that is passed around the apex of the heart and then retracted toward the base. Finally it can be tightened at the atrioventricular groove. After the procedure, the right atrial appendage must be closed with a percutaneous occluder device.

The device has not yet been implanted in humans but it was applied in 16 Yorkshire swines, including 4 with functional TR. Tricuspid septal-lateral and anteroposterior dimensions, the annular area and perimeter was significantly reduced. Small postprocedural effusions (mean, 46 ml) resolved completely at follow-up. There is no other trial registered at Clinical Trials.

##### 6.3.2.4. Cardioband

The Cardioband repair system (Edwards Lifesciences) is an approved treatment of secondary (functional) mitral regurgitation (FMR). It has been recently used in a clinical trial in patients with severe tricuspid regurgitation caused by a dilatation of the right annulus. The cardioband system is a catheter-based device that functions as a percutaneous annuloplasty band using a transfemoral approach. Implantation of the Cardioband is performed by stainless steel anchors (6 mm long). After cinching of the Cardioband the device reduces the tricuspid annular dimensions. Studies are currently investigating the use of the Cardioband in tricuspid regurgitation (NCT02981953, NCT03382457).

##### 6.3.2.5. Millipede IRIS Transcatheter Annuloplasty Ring

The IRIS is a complete semi-rigid annuloplasty ring that is placed via a transfemoral venous approach. After puncture of the femoral vein a delivery catheter is advanced into the right atrium. The delivery catheter places the device supra-annularly and the ring is then anchored and cinched reducing annular size and therefore valvular regurgitation. Up to date annuloplasty device is only studied in patients with mitral regurgitation (NCT02607527).

#### 6.3.3. Valve Replacement

##### 6.3.3.1. Orthotopic Valve Replacement

The tricuspid annulus is a complex and highly dynamic structure offering little resistance for long-term fixation of orthotopic valves. Particularly under conditions of severe TR the annulus is massively dilated with a loss of anatomic landmarks. Therefore, the development of transcatheter orthotopic valve implantation is associated with specific challenges, including device fixation, paravalvular sealing, sizing and thrombogenicity in a low-flow, low-pressure circulation. The NaviGate transcatheter tricuspid valve has been developed for orthotopic correction of severe functional TR by “NaviGate Cardiac Structures Inc.. The bioprosthetic self-expanding valve with a size of up to 52 mm has been implanted successfully in compassionate cases (50).

##### 6.3.3.2. Heterotopic Valve Replacement

**Figure 2:**
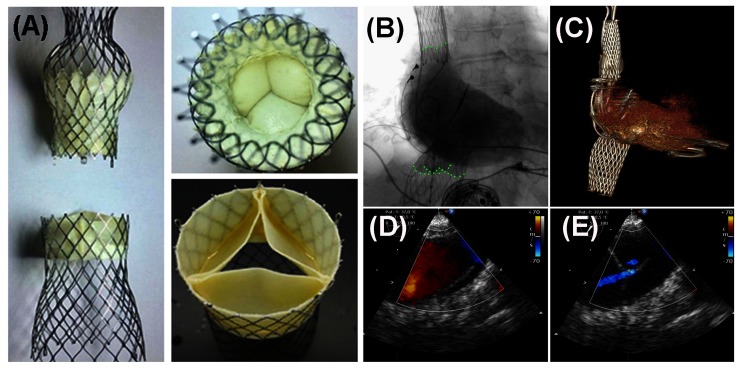
Caval Valve Implantation (CAVI) using balloon-expandable valves (SEV). **(A)** The TricValve-SEV, an investigational device with two designated valves for SVC and IVC position has been used for CAVI. **(B, D, E)** An angiogram of the right atrium and transesophageal echo demonstrates function of both valves. **(C) **Position of both valves is visualized by CT.

Severe TR leads to a systolic backflow in the hepatic, abdominal and peripheral veins causing peripheral oedema, liver cirrhosis, ascites and gastrointestinal dysfunction. Implanting a prosthetic valve in the central venous system (caval valve implantation = CAVI) reduces the venous congestion leading to amelioration of peripheral signs of right heart failure (51). Heterotopic valve implantation can be performed as single valve in the IVC or as dual valve in the IVC and SVC. The major challenges to valve implantation are the variable and large diameter of the vena cava inferior and superior and the short distance between the right atrium to the hepatic veins. “First-in-man” implantation of a self-expanding customized prosthetic valve in the IVC was reported in 2011 (51). The patient was a 79-year-old female with severe functional TR considered inoperable due to multiple previous open-heart surgeries. The patient had suffered from progressive symptoms of RV congestion with peripheral oedema, liver cirrhosis, and persistent ascites with gastrointestinal dysfunction. Procedure was performed under general anesthesia in a hybrid operating room. The prosthetic valve was delivered from the right femoral vein with a custom-made 27 F implantation catheter. After satisfactory position was confirmed by fluoroscopic and echocardiographic visualization valve was released from catheter. There were no intra- and postprocedural complications. After deployment, excellent function of the device was observed and echocardiography showed a nearly abolished ventricular wave after valve deployment. During follow-up the patient experienced a gradual improvement of symptoms related to venous congestion and right heart failure. Despite the promising data from compassionate use of the CAVI procedure data demonstrating clinical efficacy is still lacking. A recent observational trial in 25 patients demonstrated the safety and hemodynamic efficacy of CAVI using either the IVC-only or the BiCAVI approach (52). For CAVI only a few valves are suitable. The percutaneous valve system (Tric Valve, P&F GmbH, Vienna, Austria) has been developed for the bicaval venous implantation (Figure 2). It is composed of two separate three leaflet pericardial tissue valves reaching from 28 to 43 mm. Beside the Tric Valve balloon-expandable valves (BEV) like the commercial available Edwards Sapien XT and Sapien 3 can be used. These valves are designed for treatment of aortic stenosis (29 mm Edwards Sapien XT or Sapien 3; Edwards Lifesciences, Irvine, CA), but there is a growing experience in the off-label use of these devices for treating severe TR. Because of the large diameter of the cavoatrial junction, the inflow of hepatic veins and the compliance of the venous wall direct implantation of a BEV and requires the preparation of a landing zone by implanting a self-expandable stent to facilitate valve fixation. A CAVI using BEV is mostly performed only in the IVC but it can also be implanted in the IVC and SVC as a BiCAVI approach. After implantation of a heterotopic valve in the low-pressure system a lifelong anticoagulation will be required irrespective of the valve type.

## 7. Conclusion

The management of patients with severe symptomatic TR remains challenging. As patients are frequently referred for surgery late in the disease process this treatment is associated with an excessive morbidity and mortality. On the other hand, medical therapy, consisting primarily of escalating doses of diuretics is frequently ineffective as patients develop increasing diuretic resistance secondary to worsening renal function. Therefore, interventional treatment approaches are needed and multiple devices are in preclinical and clinical stages of investigation.

## Author Contributions

All authors listed, have made substantial, direct and intellectual contribution to the work, and approved it for publication.

## Conflict of Interest Statement

The authors declare that the research was conducted in the absence of any commercial or financial relationships that could be construed as a potential conflict of interest.
